# CAR‐T Cell Therapy in Autoimmune Setting: A New Appealing Approach Extendable to Allergy?

**DOI:** 10.1111/all.16585

**Published:** 2025-05-06

**Authors:** Federico Rossi, Rubén Fernandez Santamaria, Riccardo Castagnoli

**Affiliations:** ^1^ Pediatric Unit, Department of Clinical, Surgical, Diagnostic, and Pediatric Sciences University of Pavia Pavia Italy; ^2^ Pediatric Clinic Fondazione IRCCS Policlinico San Matteo Pavia Italy; ^3^ Immunology Department IIS‐Fundacion Jimenez Diaz, Hospital Universitario Fundación Jiménez Díaz Madrid Spain

**Keywords:** allergy treament, autoimmunity, B cells, cellular therapy, clinical immunology, personlized medicine, precision medicine, T cells

**Abbreviations**
CAR: chimeric antigen receptor
CNS: central nervous system
IIM: idiopathic inflammatory myositis
Ig: immunoglobulin
SLE: systemic lupus erythematosus
SSc: systemic sclerosis

Autoimmune diseases have always been a great challenge in biomedical research and patient management, since organ damage and high mortality rates remain substantial issues to address.

Current strategies involve immunosuppressive treatments, often unable to achieve a remission, and B‐cell targeting therapies, such as monoclonal antibodies, which have shown some efficacy, but they still have not enabled drug‐free and long‐term remission.

Recently, chimeric antigen receptor (CAR) T‐cell therapy has become a powerful therapeutic strategy, especially for hematologic malignancies, by targeting B cells [1]. Thus, since autoimmunity is often driven by autoreactive B cells, CAR‐T therapies could also be used to treat autoimmune diseases.

In this regard, Schett and colleagues have evaluated CD19 CAR T‐cell therapy in eight patients with systemic lupus erythematosus (SLE), three with idiopathic inflammatory myositis (IIM), and four with systemic sclerosis (SSc) (Figure 1). All of them had previously failed multiple immunosuppressive treatments [2]. Following CAR T‐cell infusion, the cells have expanded rapidly in vivo, leading to B‐cell depletion within a week. No significant adverse effects have occurred, and 11 of the 15 patients experienced a mild cytokine release syndrome.

**FIGURE 1:**
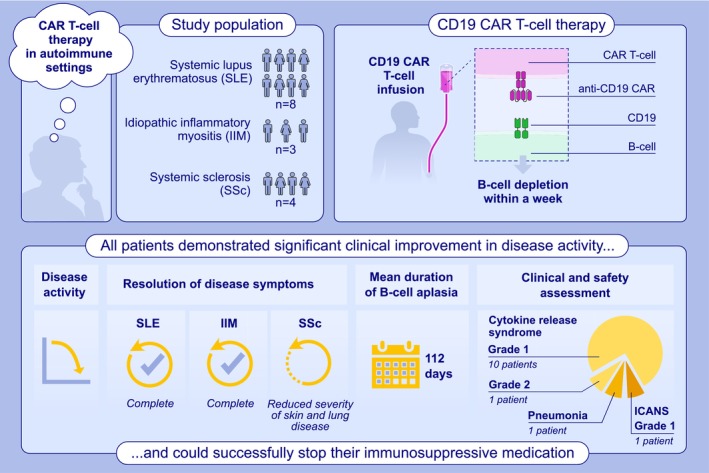
CAR‐T cell in autoimmune settings. B‐cell depletion promoted significant and sustained clinical improvement in all 3 settings. CAR‐T cells, chimeric antigens receptors T cells; IIM, idiopathic inflammatory myositis; SLE, systemic lupus erythematosus; SSc, systemic sclerosis.

After the swift depletion, the B‐cell reconstitution has occurred after a mean of 3.5 months (112 days) with a concurrent reshape of the repertoire towards a naïve B‐cell phenotype. Interestingly, even after the profound B‐cell depletion, protective Immunoglobulin G (IgG) responses to standard vaccines have remained stable, and only a moderate decrease in antibodies against pneumococci and severe acute respiratory syndrome coronavirus 2 (SARS‐CoV‐2) has been noted in the long‐term observation. Nevertheless, infections have been the main concern after the B‐cell depletion, but only one patient was admitted to the hospital for treating pneumonia.

Finally, and most importantly, all patients demonstrated significant clinical improvement in disease activity, discontinuing glucocorticoids and all the other immunosuppressive medications by the time of the final follow‐up. Moreover, the clinical improvement has also been followed with a normalization of the biomarkers in SLE (anti‐dsDNA antibodies, complement C3 levels, and proteinuria) and IIM (creatine kinase) patients, which have been maintained negative throughout the entire follow‐up. Moreover, the same group [3] has proved that using this therapeutic approach can be feasible even with SLE involving the central nervous system (CNS): CAR‐T cells can enter the CNS and precisely remove all the autoreactive B cells and, as such, induce a rapid clinical improvement.

In addition to their application in autoimmune diseases, CAR‐T therapies could also be exploited by targeting specific key cell populations in severe atopic allergic diseases, paving the way for a new application of this therapeutic tool.

Recently, some groups have shown promising results in vitro and in murine animal models by targeting B cells [4] and eosinophils [5].

In summary, CAR T‐cell therapy could represent a new therapeutic option for refractory autoimmune and severe allergic diseases, offering the potential for long‐lasting remission with manageable safety concerns. Nevertheless, further studies are needed to understand the feasibility and the potential long‐term side effects of this innovative therapeutic approach.

## Author Contributions

All authors approved the final version of the manuscript as submitted and agreed to be accountable for all aspects of the work.

## Conflicts of Interest

The authors declare no conflicts of interest.
